# Exploration of tumor-suppressive microRNAs silenced by DNA hypermethylation in cervical cancer

**DOI:** 10.1186/1743-422X-10-175

**Published:** 2013-06-03

**Authors:** Tingting Yao, Qunxian Rao, Longyang Liu, Chengyu Zheng, Qingsheng Xie, Jinxiao Liang, Zhongqiu Lin

**Affiliations:** 1 Department of Gynecological Oncology, Sun Yat-sen Memorial Hospital, Sun Yat-sen University, 107 Yan Jiang West Road, Guangzhou 510120, People’s Republic of China; 2 Key Laboratory of malignant tumor gene regulation and target therapy of Guangdong Higher Education Institutes, Sun Yat-sen University, 107 Yan Jiang West Road, Guangzhou 510120, People’s Republic of China

**Keywords:** Cervical cancer, Hypermethylation, miRNA

## Abstract

**Background:**

Multiple studies proved that miRNAs have a causal role in tumorigenesis. Some miRNAs are regulated by epigenetic alterations in their promoter regions and can be activated by chromatin- modifying drugs.

**Methods:**

We treated cervical cancer cells with 5-aza-2’-deoxycytidine and get a microarray analysis. Dysregulation of miRNAs was measured by qPCR in cervical cell lines and methylation status of them in cervical cancer tissue were performed with MeDIP-qPCR assay.

**Results:**

We found hypermethylation of miR-432, miR-1286, miR-641, miR-1290, miR-1287 and miR-95 may have some relationship with HPV infection in cervical cell lines. In primary tumors of cervix with paired normal tissue, expression levels of miRNAs were inversely correlated with their DNA methylation status in the cervical cancer cell lines treated with 5-AZA.

**Conclusions:**

Our results indicate that miRNAs might play a role in the pathogenesis of human cervical cancer with HPV and identify altered miRNA methylation as a possible epigenetic mechanism involved in their aberrant expression.

## Introduction

Cervical cancer is the second most common cancer in women worldwide. Approximately 80% of primary cervical cancers arise from pre-existing squamous dysplasia. The most important etiologic agent in the pathogenesis is human papillomavirus (HPV). However, not all women infected with high-risk HPV develop cervical carcinoma. It is obviously that many genetic and epigenetic alternations occur during its tumorigenesis. Among those changes, aberrant promoter methylation of tumor-suppressor genes gives rise to its silencing functions and results in the significant carcinogenesis of cervical cancer.

Until recently, few evidences have shown that miRNA genes may be regulated by epigenetic mechanisms, as changes in genomic DNA methylation pattern. In this study, we present the results of a genome-wide miRNA expression in cervical cancer cells treated with 5-aza-2’-deoxycytidine and identify the altered methylation of miRNA genes as a possible epigenetic mechanism responsible for their aberrant expression.

## Material and methods

### Cell culture

Four human cervical carcinoma cell lines (C33A, Hela, CaSki and SiHa) were obtained from the American Type Culture Collection (Manassas, VA) and cultured in DMEM supplemented with 10% fetal bovine serum (Invitrogen) at 37°C and 5% CO_2_ in a humidified incubator. The Caski line was reported to contain an integrated human papillomavirus type 16 genome (HPV-16, about 600 copies per cell) as well as sequences related to HPV-18(Schache AG, et al., [1]). The Siha line was reported to contain an integrated human papillomavirus type 16 genome (HPV-16, 1 to 2 copies per cell) (Bishop JA, et al., [2]). Hela cell contains an integrated HPV-18 genome (Iwakawa R, et al., [3]), while C33A contains no HPV (Hwang CF, et al., [4]).

### Clinical samples

Fifty histopathologically verified cervical tumor samples and matched normal tissues were obtained from patients, who were admitted to the Department of Gynecologic Oncology of Sun Yat-sen Memorial Hospital, Sun Yat-sen University. The tissues were snap frozen in liquid nitrogen immediately after surgical removal and stored at −80°C until required. None of the patients recruited into the present study received radiotherapy or chemotherapy. All of them were tested using the HybriBio Human Papillomavirus GenoArray Test Kit (Liu SS, et al., [5]) and infection of HPV-16 or 18 was confirmed upon hospital admission. Adjacent normal tissue samples were taken at least 1 cm distal to tumor margins.

### Demethylating experiment

Cervical cancer cells (2×10^4^/well) were allowed to grow in 6-well plates 48 h before treatment with 10 μmol/L 5-aza-2’-deoxycytidine (5-AZA; Sigma). After 24 h of treatment, cells were collected and total RNA was isolated using Trizol reagent. Three replicates for both untreated cells and 5-AZA–treated cells were used to evaluate the miRNA expression by microarray profiling. Differentially expressed miRNAs were identified by using univariate two-class *t* test with random variance model.

### miRNA microarray hybridization and quantification

Total RNA isolation was done with Trizol (Invitrogen) according to the manufacturer’s instructions. Using miRCURY LNA Array (version 11.0) system, RNA samples were labeled with the Exiqon miRCURY Hy3/Hy5 power labeling kit and hybridized on the miRCURY LNA Array (version 11.0) station. Hybridization signals were detected with streptavidin-Alexa Fluor 647 conjugate and scanned images (Axon 4000B) were quantified using the GenePix 6.0 software (Axon Instruments) to read image raw intensity. The intensity of the green signal was calculated after background subtraction, and replicated spots on the same slide were averaged to obtain median intensity. The median normalization method was used to acquire normalized data (foreground minus background divided by median). The median was the 50th percentile of miRNA intensity and was >50 in all samples after background correction. The threshold value for significance used to define upregulation or downregulation of miRNAs was a fold change >1.5, with a value of *P*<0.05 calculated by the *t* test. Differentially expressed miRNAs were identified by using the *t* test procedure within significance analysis of microarrays (SAM) (Tusher VG, et al., [6]). The miRNAs selected for investigation in our study were further filtered on the basis of expression levels and previously published data.

### Quantitative real-time PCR

The RT reaction used MMLV reverse transcriptase (Epicenter, Madison, WI). Quantitative PCR was performed by an ABI PRISM7500 system (Applied Biosystems, Foster City, CA). Selected miRNAs were further quantified with TaqMan quantitative RT-PCR.All reactions were done in a 20 μL reaction volume in triplicate by SYBR Green Real-time PCR Universal Reagent (GenePharma Co., Ltd.) and analyzed by MX-3000P Real-time PCR machine (Stratagen). Standard curves were generated and the relative amount of miRNA was normalized to U6 snRNA (2^−ΔCt^). Expression fold-change was evaluated using 2^−ΔΔCt^. Let-7a was used as control. Primers were as follows (Additional file 1: Table S1).

### Methyl-DNA immunoprecipitation–qPCR

MiRNAs from patients were prepared by overnight proteinase K treatment, phenol–chloroform extraction, ethanol precipitation, and RNase digestion. Following denaturation, miRNAs were incubated overnight at 48°C with 8 micrograms of 5-methylcytidine monoclonal antibody (Eurogentec). Fifty microliters of rabbit anti-IgG magnetic beads (BioLabs S1430S) was added and incubated for 2 h at 48°C,. Magnetic beads–monoclonal antibody complexes were sequentially washed by gentle mixing at 48°C for 4 min with 1 microgram of wash buffer 1 (2 mM EDTA, 20 mM Tris [pH¼8.0], 1% Triton X- 100, 0.1% sodium dodecyl sulfate [SDS], 150mMNaCl), wash buffer 2 (2 mM EDTA, 20 mM Tris [pH¼8.0], 1% Triton X- 100, 0.1% SDS, 500 mM NaCl), and wash buffer 3 (1 mM EDTA, 10 mM Tris [pH¼8.0]). After washing, the complexes were subjected to magnetic separation rack for 10 min at 48°C, and then elution was performed with 400 microlitre elution buffer (50 mM Tris–HCl [pH 8.0], 10 mM EDTA [pH 8.0], 1% SDS). The elution fraction was subjected to phenol–chloroform extraction and ethanol precipitation. The quantity of immunoprecipitated miRNA was checked with a Nanodrop spectrophotometer (Agilent) (Liu BL et al., [7]; Zhang Y, et al., [8]). PCR amplification using the real-time PCR was performed as described above. Relative enrichment of miRNA methylation for each gene was determined by the same method described above. The PCR primers used for each gene in this analysis are given in Additional file 2: Table S2.

### Ethics approval

All database searches were carried out by Ethics committee of Sun Yat-sen Memorial Hospital, Sun Yat-sen University(IRB of Sun Yat-sen Memorial Hospital 2012[03]). Patient data were kept to a minimum and stored in a secure manner on a database under the control of the University of Sun Yat-Sen to which only the corresponding author has access.

### Statistical analysis

Quantitative data are shown as mean values standard deviation. Statistical analyses were performed using independent-samples t-test. *P* <0.05 was regarded as statistically significant.

## Results

### miRNA microarray analysis

**Figure 1:**
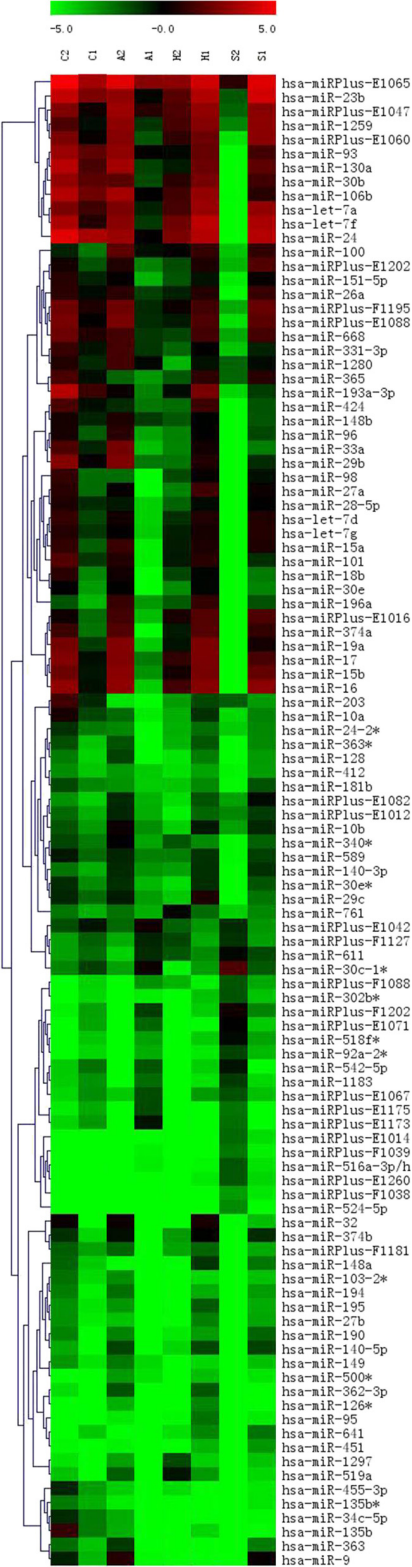
**Hierarchical cluster tree of representation miRNAs in cell lines before and after treatment with the demethylating agent 5-AZA.** Each row represented a miRNA and each column represented a sample. The miRNA clustering tree was shown on the left, and the sample clustering tree appeared at the top. The color scale shown at the top illustrated the relative expression level of a miRNA: red color represented a high expression level; green color represented a low expression level. The clustering was performed on differentially expressed miRNAs (differentially expressed in all 4 comparisons); the low intensity expressed miRNAs were filtered (which ForeGround- BackGround intensities were all <50 in all the samples). (A1: C33A treated with 5-AZA;A2: C33A; C1: Caski treated with 5-AZA; C2: Caski; H1: Hela treated with 5-AZA; H2: Hela; S1: Siha treated with 5-AZA; S2:Siha).

Hierachical clustering analyses of miRNA expression showed significant changes (>1.5-fold difference) in expression levels (the resulting hierarchical cluster tree is reported in (Figure 1). The heat map diagram showed the result of the two-way hierarchical clustering of genes and samples. Compared with control, there were 172 miRNAs upregulated and 192 miRNAs down-regulated in C33A, 40 miRNAs upregulated and 138 miRNAs down-regulated in Caski, 207 miRNAs upregulated and 103 miRNAs down-regulated in Hela, 231 miRNAs upregulated and 271 miRNAs down-regulated in Siha (Additional file 3: Table S3 and Additional file 4: Table S4). MiR-432, miR-1286, miR-641, miR-1290, miR-1287 and miR-95 were found up-modulated in Caski, Hela and Siha but not in C33A induced on treatment, while miR-625 was down-modulated.

**Figure 2:**
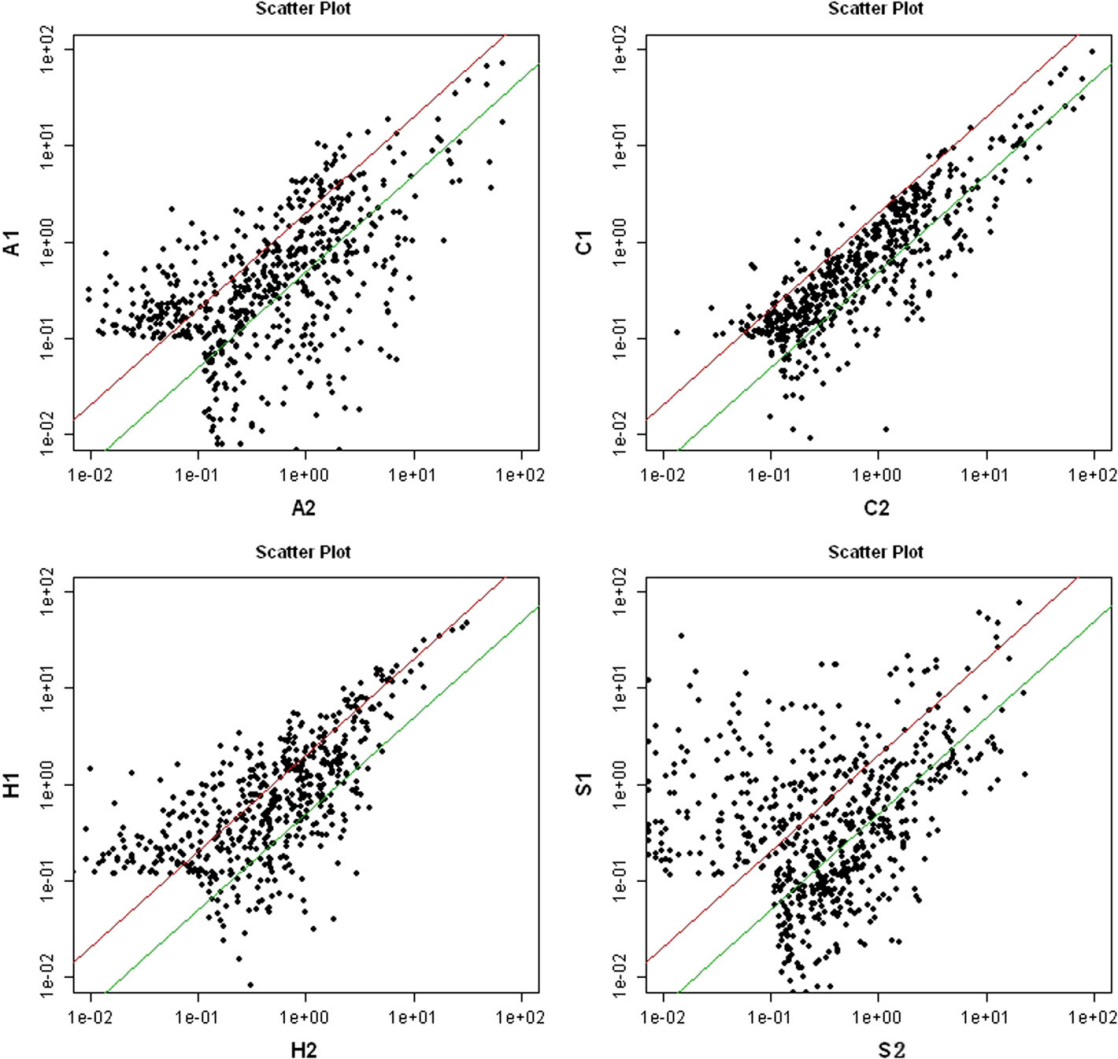
**Correlation R-value in the Correlation & Scatter Plot.** The data represented the change of miRNA profiling before (x axis) and after (y axis) the 5-AZA treatment in different tumor cells (Up left: C33A, Up right: Caski, low left: Hela, low right: Siha). The red lines in the panals represented the threshold of upregulation,while green lines represented the threshold of downregulation of miRNA expression.

The *R*-value was calculated after array normalization using the Median method. When two samples were different, the correlation coefficient *R* of them did not mean the reproducibility of the slides but the difference of samples. When two samples were the same, the correlation coefficient *R* of them reflected the reproducibility of the slides. The correlation *R*-value was shown in the Correlation & Scatter Plot sheet (Figure 2) which showed the difference between untreated and treated arrays.

### Validation of miRNA expression

**Figure 3:**
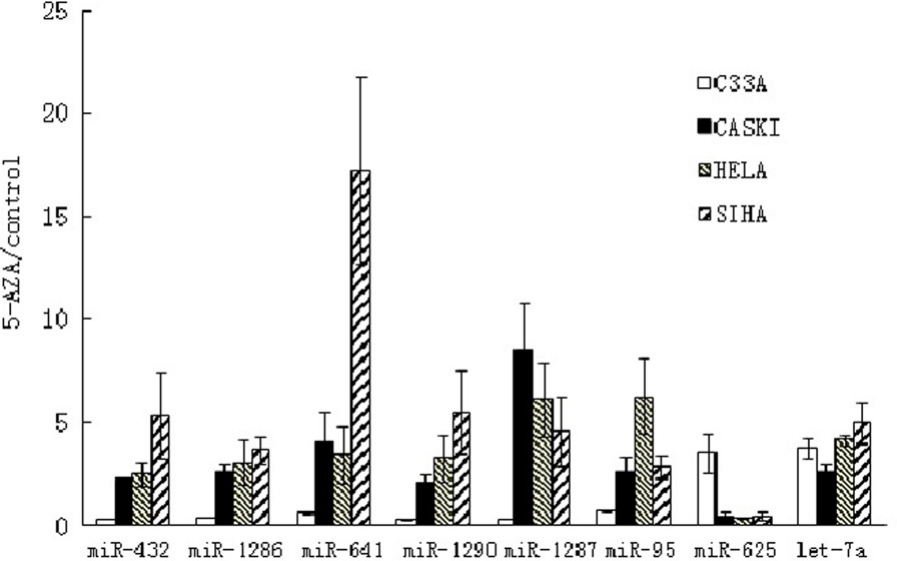
Real-time PCR to verify the disregulated miRNAs founded in Caski, Hela and Siha groups.

These miRNAs whose expression was significantly dysregulated based on microarray analysis were selected for confirmation by qRT-PCR. Methylation of let-7a has been reported before (Lu L, et al., [9]), so it was chosen as control. We found that miR-432, miR-1286, miR-641, miR-1290, miR-1287 and miR-95 were highly upregulated just in cells with HPV infection upon 5-AZA treatment, whereas miR-625 was significantly downregulated (*P*<0.05) (Figure 3). Therefore, the analysis of selected miRNAs by qPCR confirmed the microarray results.

**Figure 4:**
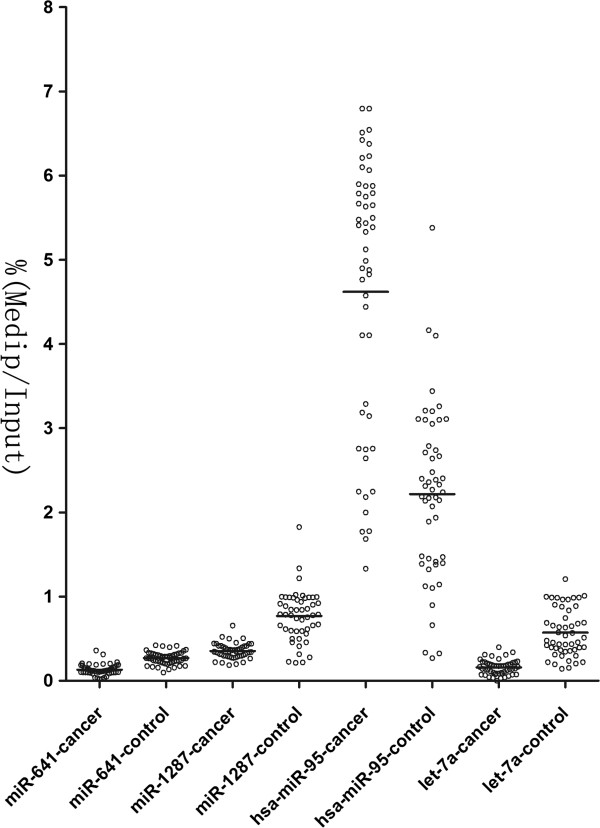
**Methylation status of disregulated miRNAs in cervical cancer tissue were performed with MeDIP-qPCR assay.** Calculation of the MeDIP gene of interest/input gene of interest ratios was based on the fluorescence emission intensity values. Black bars denote the calculated cutoff values.

MiR-641, miR-1287 and miR-95 changing most significantly in cell lines were chosen for further study in cervical tissue. Methylation status in cervical cancer tissue were performed with MeDIP-qPCR assay (Figure 4) compared with adjacent normal tissue. Interestingly, expression of miR-641 and miR-1287 in cervical tissue was lower than control, while miR-95 was higher (*P*<0.05).

## Discussion

MicroRNA (miRNA) is a novel class of short non-coding RNA molecules regulating a wide range of cellular functions through translational repression of their target genes. DNA methylation is one of the heritable epigenetic modifications, repressing gene expressions and consequent phenotypic alterations without changing the DNA sequence. Recently, epigenetic dysregulation of tumor suppressor miRNA genes by promoter DNA methylation has been implicated in human cancers.

In our previous study, we found miR-21 is significantly overexpressed in human cervical squamous cancer tissues and cell lines. We also proved that miR-21 is involved in cervical squamous cell tumorigenesis by CCL20 (Yao T, et al., [10]). 5-Aza-CdR which can reactivate the expression of methylated tumor suppressor genes, is a pyrimidine nucleoside analog that forms a covalent complex with DNMT during DNA replication (Yao TT, et al., [11]). To evaluate if an aberrant DNA methylation pattern could also contribute to the altered miRNA expression characterizing the cervical carcinoma, we analyzed the miRNA profiling of the cervical cancer cell lines before and after treatment with 5-AZA. Resulting from our analyses, a number of miRNAs were over-expressed but not reported, as well as down-modulated but not deleted. The involvement of an epigenetic regulatory mechanism could actually exert a role on miRNA expression in human cervical cancer.

It is conceivable that several disregulated miRNAs with no defined functions at this point may exhibit tumor suppressor or oncogene activity and normally target components of key signaling pathways that promote and maintain the growth and survival of cells. Several analysis revealed high expression level of miR-432 which remained significant as independent predictor for recurrence-free (RFS) of hepatocellular carcinoma (Huang YH, et al., [12]). However, in human pituitary GH adenomas, miR-432 plays a role as tumor suppressor gene by regulating HMGA2 (D’Angelo D, et al., [13]).

Thirteen miRNAs including of miR-1286 were deregulated in both two esophageal carcinoma cell lines (one adenocarcinoma and one squamous cell carcinoma) treated with cisplatin or 5-fluorouracil for 24 or 72 h (Hummel R, et al., [14]). Analysis of predicted targets of it highlighted molecular networks included functions such as ‘Cell death’, ‘Cell cycle’, ‘Cellular growth and proliferation’, ‘DNA replication, recombination, and repair’ and ‘Drug metabolism’. Cisplatin or 5-fluorouracil altered miRNA expression in esophageal cancer cells. Ingenuity Pathway Analysis (IPA) suggested that miR-1286 may target molecular pathways involved in cell survival after chemotherapy.

Until recently, there has been just one report about miR-641 up-regulated in normal chondrocytes (Díaz-Prado S, et al., [15]). *In silico,* analyses predicted that key molecular pathways potentially altered by miRNAs differentially expressed in normal and OA chondrocytes include TGF-beta, Wnt, Erb and mTOR signalling; all of them implicated in the development, maintenance and destruction of articular cartilage. We concluded the up-modulation of miR-641 in our study may also have relationship with these signaling.

Researches on miR-1290 have been referred on tumor therapy. Following in vitro photodynamic treatment, miR-1290 was overexpressed in human epidermoid carcinoma cells (A431) (Bach D, et al., [16]). Up-regulation of microRNA-1290 impairs cytokinesis and affects the reprogramming of colon cancer cells (Wu J, et al., [17]). In light of these reports and our results, we proposed miR-1290-based therapeutic approaches could be developed in future.

Wilcoxon sign-rank test for paired samples analysis revealed that abnormal miRNAs showed a higher level of variation across different breast cancer samples. Most of these miRNAs were significantly down-regulated in tumor samples, but miR-1287 was consistently expressed in tumor tissues and serum samples (Guo L, et al., [18]). In follicular lymphoma, it was increased (Wang W, et al., [19]).

MiR-95 has been shown to be involved in carcinogenesis. A highly characterized example is pancreatic cancer, in which miR-95 was confirmed significantly increasing cell proliferation, invasion, migration and inhibited cell apoptosis in vitro and in vivo when compared with negative control (Li WG, et al., [20]). Concordant changes were also revealed in colorectal carcinoma (Huang Z, et al., [21]) and pancreatic cancer (Zhang Y, et al., [22]). However in head and neck cancers, miR-95 has been suggested downregulated (Nurul-Syakima AM, et al., [23]). In HeLa cells, inhibition of miR-95 caused a decrease in cell growth (Cheng AM, et al., [24]).

In our study, we found miR-625 was decreased after demethylation. Increasing evidence points to a central role of miR-625 for vesicle trafficking processes in oncogenesis and tumor suppression. Expression of miR-625 is significantly down-regulated and negatively correlated with lymph node metastasis in gastric cancer. MiR-625 significantly inhibits invasion and metastasis of gastric cancer cells both in vitro and in vivo. ILK is a direct target gene for miR-625 and knockdown of ILK has a phenocopy of overexpression of miR-625 (Wang M, et al., [25]). In the hormonal treatment of endometrial carcinoma, Hec1A cells were treated with medroxyprogesterone acetate, the expression levels of miR-625 was increased by more than 400% (Bae J, et al., [26]).

The methlyation DNA immunoprecipitation-based chip analysis (MeDIP-chip) is a novel high- throughput array-based method using a monoclonal antibody against 5-methycytidine for the enrichment of the methylated DNA fragments and then hybridizing to the promoter and CpG islands of the entire human genome (Zhang Z, et al., [27]). Our study found that the contents of promoter methylation in miR-641 and miR-1287 were inversely correlated with the expression levels of these genes in cells treated with 5-aza-2’-deoxycytidine. Contradictorily, our results showed that after treatment of 5-AZA, miR-95 was significantly up-regulated in cell lines, whereas the expression of it was lower in cervical cancer than normal tissues. We proposed there may be some other factors coexisted.

However, these different human miRNAs were not found in microarray of C33A. So, we concluded that the downregulation may have relationship with the negative expression of HPV in C33A. It is intriguing to speculate that the expression of cellular miRNA genes at or near HPV integration sites may contribute to the tumor phenotype.The involvement of an epigenetic regulatory mechanism could actually exert a role on miRNA expression in cervical cancer. Apparently, our findings may provide new insights into understanding the pathogenesis of cervical cancer.

## Competing interests

The authors declare no conflict of interest.

## Authors’ contributions

ZL was responsible for study design. MY was responsible for the experiment, data analysis, literature search and article drafting. Other authors were responsible for histopathological information collection of all data. All authors read and approved the final manuscript.

## Supplementary Material

Additional file 1: Table S1 Primers of real-time PCR. Click here for file

Additional file 2: Table S2 Primers of Medip-qPCR. Click here for file

Additional file 3: Table S3 The up-regulated microRNAs after 5-Aza treatment. Click here for file

Additional file 4: Table S4 The down-regulated microRNAs after 5-Aza treatment. Click here for file
